# ERCP Features and Outcome in Patients with Periampullary Duodenal Diverticulum

**DOI:** 10.1155/2013/217261

**Published:** 2013-07-28

**Authors:** Amir Houshang Mohammad Alizadeh, Esmaeil Shamsi Afzali, Anahita Shahnazi, Mirhadi Mousavi, Siavash zafar Doagoo, Dariush Mirsattari, Mohammad Reza Zali

**Affiliations:** Gastroenterology and Liver Diseases Research Center, Shahid Beheshti University of Medical Sciences, Tehran 19857, Iran

## Abstract

*Background*. Although periampullary diverticulum is usually asymptomatic and discovered incidentally in patients during endoscopic retrograde cholangiopancreatography (ERCP), it may lead to post-ERCP morbidity. We compared baseline characteristics and clinical data as well as ERCP results in patients with and without periampullary diverticulum. *Methods*. Clinical, laboratory, and ERCP data of 780 patients referred to the Taleghani Hospital, as a great referral endoscopy center, in Iran were prospectively analyzed. *Results*. The periampullary diverticulum was identified in 44 patients (5.6%). Cannulation of common bile duct was more failed in patients with diverticulum compared to others (35.5% versus 11.5, *P* < 0.001). Patients with diverticulum had eight times more often common bile duct stone compared to patients without diverticulum (54.5% versus 12.2%, *P* < 0.001). Post-ERCP complications were observed in 2.3% and 4.2% of patients with and without diverticulum, respectively, which did not significantly differ in both groups. *Conclusion*. Because of more failure cannulation in the presence of periampullary diverticulum, ERCP requires more skills in these patients. Prevalence of common bile duct stone was notably higher in patients with diverticulum; therefore, more assessment of bile stone and its complications in these patients is persistently recommended.

## 1. Introduction

Periampullary diverticulum is commonly situated on the second part of the duodenum and usually occurred in the elderly, with a slight female preponderance [1, 2]. It is usually caused by the progression of duodenal motility disorders. Furthermore, progressive weakening of intestinal smooth muscles and increased intraduodenal pressure have been known as main underlying etiologies for this defect [3]. The incidence of this finding widely varies from 1% to 32.8% based on different diagnostic approaches such as barium graphs, endoscopic retrograde cholangiopancreatography (ERCP) evaluation, and autopsy series [4–7]. Although periampullary diverticulum is usually asymptomatic and discovered incidentally in patients during ERCP, its association with various pathological conditions such as choledocholithiasis, perforation, pancreatitis, bleeding, CBD obstruction, and rarely carcinoma has been well recognized in various studies [8–12]. One of the most important problems to the endoscopists is the impact of these diverticula on the success of therapeutic or diagnostic ERCP procedures. In some reports, cannulation difficulty during ERCP was associated with the presence of periampullary diverticulum [3] and, in some others, it was related to higher risk of retained stones in the common bile duct [5]. However, some other studies could not find a relationship between the presence of diverticulum and any technical difficulties at ERCP. Moreover, successful cannulation was achieved in 88.8% to 97% of patients with diverticulum [2, 11, 12]. Therefore, more studies should be performed with the aim to determine the ERCP success rate and its related complications in patients with periampullary duodenal diverticulum.

We assessed and compared baseline characteristics and clinical data as well as ERCP results and complications in patients with and without periampullary diverticulum.

## 2. Materials and Methods

The study was approved by institutional review board of the Shahid Beheshti University of Medical Sciences. Patients between the ages of 15 and 99 with the diagnosis of hepatobiliary diseases and candidate for ERCP referred to Taleghani referral hospital between 2009 and 2012 were eligible and underwent diagnostic and therapeutic ERCP procedure. Patients with these criteria were ineligible: age below 15 years, acute illness such as hypotension, hypoxia, oxygen saturation less than 95% on supplemental oxygen, and hemodynamic instability. Patients with surgically altered anatomy (Billroth II or Roux-en-Yanastomosis) were also excluded as cannulation technique is then fundamentally different from that in normal anatomy.

Participants classified as one of the two groups: patients were diagnosed periampullary diverticulum following ERCP (as the case group) and those without this finding (as the reference group). Data describing patient characteristics such as demographic characteristics, medical history, and clinical presentations were collected from patients recorded files and by interviewing in the day of admission to hospital if required. Laboratory parameters were also measured in the day of admission that consisted of cell blood count and liver function tests. Eligible patients underwent ERCP for suspected and diagnosed pancreatobiliary disease on the basis of generally accepted diagnostic indications for ERCP [13]. Procedure was performed under conscious sedation with midazolam and meperidine and by a gastroenterologist. Cannulation was performed on the basis of techniques as previously described [14]. Successful cannulation was defined as free and deep instrumentation of the biliary tree and a cannulation attempt was defined as sustained contact with the cannulating device and the papilla for at least five seconds [15]. Difficult biliary cannulation was also related to the failure of biliary access despite ten minutes of attempted biliary cannulation, or more than five attempted unintentional pancreatic cannulations [16]. Post-ERCP complications include at least one of these post-ERCP pancreatitis, gastrointestinal perforation, and bleeding. 

Comparisons of categorical variables across the groups were performed using an overall chi-square test or Fisher's exact test if required, while comparisons of continuous variables were performed using a *t*-test or Mann-Whitney test. The role of the presence of periampullary diverticulum for predicting common bile duct stone and also biliary cannulation failure was assessed by linear regression analysis adjusting for confounders. Model calibration was estimated using the Hosmer-Lemeshow (HL) goodness-of-fit statistic (higher *P* values imply that the model fits the observed data better). The data analyzer was anonymous, and data collection and processing were approved by the institutional review board of the university. 

*P* values of 0.05 or less were considered statistically significant. All the statistical analyses were performed using SPSS version 15.0 (SPSS Inc., Chicago, IL, USA) and SAS version 9.1 for Windows (SAS Institute Inc., Cary, NC, USA).

## 3. Result

A total of 780 patients underwent ERCP that of these 44 patients had periampullary diverticulum. At baseline (Table 1), there was no difference in male-to-female ratio between the two study groups, and patients with diverticulum were older than the patients without this finding (66 versus 57-year old, *P* = 0.001). Regarding medical history, there were no significant differences in the overall incidence rates for hypertension, diabetes mellitus, current smoking, and family history of coronary artery disease between the patients with diverticulum and control group. Also, two groups were similar in terms of previous history of ERCP, cholecystectomy, and biliary stone. With regard to laboratory parameters (Table 2), levels of serum total and direct bilirubins as well as liver enzymes were lower in the group with diverticulum.

**Figure 1:**
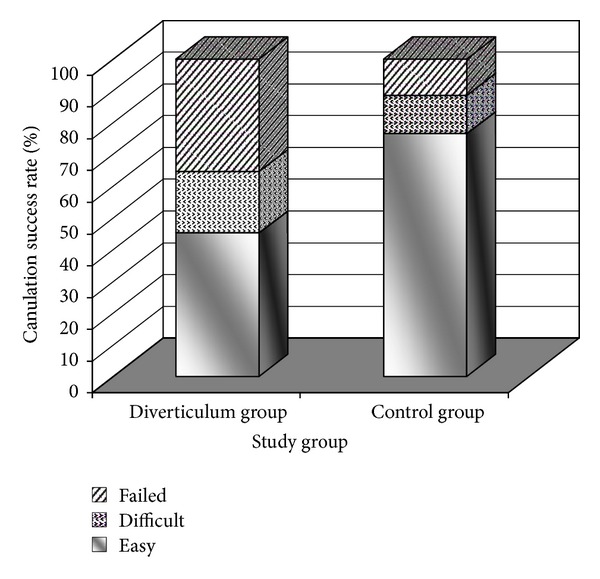
Success and failure rate of cannulation in the group with periampullary diverticulum and the control group.

Successful biliary cannulation was achieved in 64.5% of the patients with periampullary diverticulum and in 88.5% of patients without this finding (*P* < 0.001) that in 19.3% and 12.0% of them was difficulty performed, respectively (Figure 1). Cannulation of common bile duct was also more failed in patients with diverticulum compared to others (35.5% versus 11.5, *P* < 0.001). Multivariable logistic regression analysis (Table 3) also confirmed that the presence of periampullary diverticulum could predict the failed biliary cannulation (OR = 6.287, 95% CI = 2.458–16.083,  *P* < 0.001).

Those who had cannulation failure underwent needle knife precutting or fistulotomy regarding operators' preference. All of them had successful cannulation by these techniques.

In univariate analysis, patients with diverticulum had more common bile duct stone compared to patients without diverticulum (54.5% versus 12.2%, *P* < 0.001). Multivariable analysis (Table 4) also showed that the group with periampullary diverticulum had six times more often common bile duct stone in comparison with another group (OR = 6.450, 95% CI = 3.159–13.167, *P* < 0.001).

**Figure 2:**
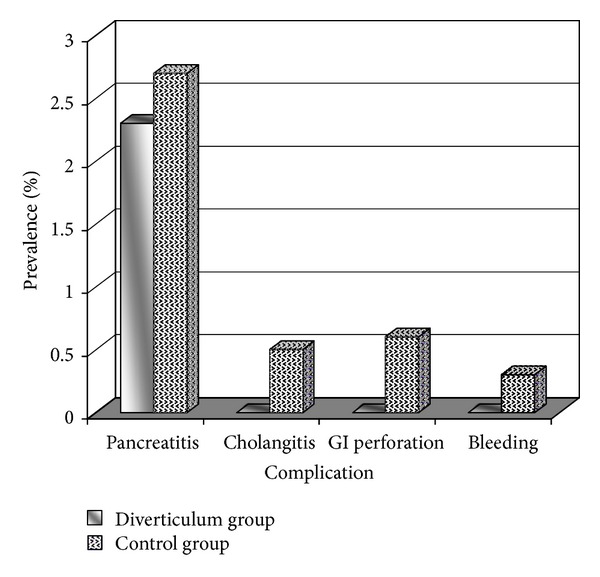
ERCP-related complications in the group with periampullary diverticulum and the control group.

 There were no significant differences between the diverticulum and control groups in term of ERCP-related complications such as pancreatitis (2.3% versus 2.7%), bleeding (0.0% versus 0.3%), gastrointestinal perforation (0.0% versus 0.6%), and cholangitis (0.0% versus 0.5%) (Figure 2).

## 4. Discussion

The current study first focused on the incidence of periampullary diverticulum among the patients who were candidate for ERCP. This phenomenon was found in 5.6% of our participants that was considerably lower than most previous studies. Some researches could confirm that high prevalence of diverticula was attributed to the higher age [2, 3, 11], and this relationship was reconfirmed in the present study since the mean age of patients was significantly higher in those with periampullary diverticulum than others (66 years versus 57 years, resp.). The discrepancy between the reported incidences of periampullary diverticulum can be also the result of differences in operator experience for detecting periampullary diverticulum. Furthermore, despite relationship of the incidence of diverticulum with female gender predominance [1, 17], this relation was not found in our study. It seems that the increased creation of periampullary diverticulum is mainly associated with advanced age, whereas its higher prevalence among women may not be reported in all studies among different population. 

We showed that the presence of diverticulum significantly increased the difficulty and failure of biliary cannulation. Similarly, in a study by Lobo et al., diverticulum was a major cause of failed ERCP, especially in patients with intradiverticular papillae in comparison with juxtapapillary diverticula [3], whereas, in some other studies, the finding of a periampullary diverticulum during an ERCP was suggested as an indicator of an easier cannulation attempt [11, 12]. The various techniques for cannulation can be responsible for explaining higher cannulation failure rate. A low cannulation rate can be also attributed to the inability of the endoscopist to detect the papilla in a substantial percentage of cases with duodenal diverticula. Also, when the papilla is located deep inside the diverticulum, often lying at the bottom, the cannulation used to be difficult.

In the present study, the group with periampullary diverticulum had six times more often common bile duct stone in comparison with another group. It is similar other studies evaluated periampulary diverticula. Kennedy and Thompson indicated that patients with biliary stone were 2.6 times more likely to have a periampullary diverticulum than patients without this finding [18]. Rajnakova et al. also found that patients with diverticulum presented 1.8-times more often with retained stone in the common bile duct than patients without diverticulum [5]. Moreover, Tham and Kelly found bile duct stones in 64% of patients with diverticula compared with 33% of the controls, with an odds ratio of 3.6 [6]. Formation of biliary stone in the presence of periampullary diverticula can be related to several probable hypotheses. First, it has been proposed that the dysfunction in the sphincter of Oddi, which in turn causes reflux of pancreatic fluid and intestinal content that lead to biliary stone formation [7]. It has been also argued that diverticula cause spasm of the sphincter and increase biliary tract pressure. This phenomenon may produce jaundice and cholangitis as well as predispose for CBD lithiasis [19]. Also, it has been hypothesized that periampullary diverticula may cause functional biliary stasis possibly by compression of the distal part of the common bile duct that accounts for the increased incidence of pigment biliary stones [20, 21]. Although we were able to demonstrate an association between the presence of periampullary duodenal diverticulum and choledocholithiasis, pathological basis of this phenomenon is already undetermined and should be supported by further studies.

In summary, although the incidence of periampullary diverticulum in our study population is lower than most previous reports, its related female predominance as well as high cannulation failure is considerable. Because of more failure cannulation in the presence of periampullary diverticulum, ERCP requires more skills in these patients. Prevalence of common bile duct stone was notably higher in patients with diverticulum; therefore, more assessment of bile stone and its complications in these patients is persistently recommended.

## Figures and Tables

**Table 1:** Baseline characteristics and medical history in the group with periampullary diverticulum and the control group.

Characteristics	Diverticulum group (*n* = 44)	Control group (*n* = 736)	*P* value
Male/female	18/26	375/361	0.193
Age (years)	65.9 ± 16.0	57.0 ± 17.1	0.001
Medical history:			
Diabetes mellitus	8 (18.2)	81 (11.0)	0.146
Hypertension	8 (18.2)	133 (18.1)	0.985
Coronary artery disease	5 (11.4)	58 (7.9)	0.410
Cigarette smoking	4 (9.1)	93 (12.6)	0.489
Cholecystectomy	20 (45.5)	263 (35.7)	0.193
Previous ERCP	3 (6.8)	67 (9.1)	0.606
Biliary stone	4 (9.1)	76 (10.3)	0.999

Data are presented as mean ± SD or *n* (%).

**Table 2:** Pre-ERCP laboratory parameters in the group with periampullary diverticulum and the control group.

Laboratory parameters	Diverticulum group (*n* = 44)	Control group (*n* = 736)	*P* value
AST	55.5 ± 46.0	87.5 ± 92.2	0.019
ALT	61.5 ± 55.9	112.7 ± 205.9	0.015
ALP	656.0 ± 753.5	832.3 ± 795.5	0.051
Lactate dehydrogenase	435.8 ± 229.7	465.4 ± 330.1	0.812
Total bilirubin	3.2 ± 6.6	6.6 ± 9.0	0.006
Direct bilirubin	2.0 ± 4.2	3.6 ± 5.2	0.025
Serum amylase	187.2 ± 211.7	167.7 ± 356.2	0.311

Data are presented as mean ± SD.

**Table 3:** Multivariable regression analysis of the predicting effect of periampullary diverticulum on failed biliary cannulation with the presence of confounders.

Item	Multivariate *P* value	Odds ratio	95% CI	
			Lower limit	Upper limit
Presence of diverticulum	<0.001	6.287	2.458	16.083
Advanced age	0.123	0.985	0.966	1.004
Hypertension	0.201	0.634	0.315	1.275
Coronary artery disease	0.192	0.576	0.251	1.318
Cholecystectomy	0.058	1.896	0.979	3.674
Serum total bilirubin	0.056	0.972	0.943	1.001

Hosmer-Lemeshow goodness of fit: *χ* ^2^ = 6.239, *P* = 0.621.

**Table 4:** Multivariable regression analysis of the predicting effect of periampullary diverticulum on common bile duct stone with the presence of confounders.

Item	Multivariate *P* value	Odds ratio	95% CI	
			Lower limit	Upper limit
Presence of diverticulum	<0.001	6.450	3.159	13.167
Male gender	0.832	1.049	0.673	1.634
Advanced age	0.018	0.983	0.970	0.997
Serum total bilirubin	<0.001	1.081	1.035	1.129

Hosmer-Lemeshow goodness of fit: *χ* ^2^ = 4.240, *P* = 0.835.
